# Temperature and water stress during conditioning and incubation phase affecting *Orobanche crenata* seed germination and radicle growth

**DOI:** 10.3389/fpls.2015.00408

**Published:** 2015-06-03

**Authors:** Juan Moral, María Dolores Lozano-Baena, Diego Rubiales

**Affiliations:** Rubiales Laboratory, Department of Plant Breeding, Institute for Sustainable Agriculture, Consejo Superior de Investigaciones Científicas, CórdobaSpain

**Keywords:** broomrape, water potential, matric and osmotic stress

## Abstract

*Orobanche crenata* is a holoparasitic plant that is potentially devastating to crop yield of legume species. Soil temperature and humidity are known to affect seed germination, however, the extent of their influence on germination and radicle growth of those of *O. crenata* is largely unknown. In this work, we studied the effects of temperature, water potential (Ψ_t_) and the type of water stress (matric or osmotic) on *O. crenata* seeds during conditioning and incubation periods. We found that seeds germinated between 5 and 30°C during both periods, with a maximum around 20°C. Germination increased with increasing Ψ_t_ from −1.2 to 0 MPa during conditioning and incubation periods. Likewise, seed germination increased logarithmically with length of conditioning period until 40 days. The impact of the type of water stress on seed germination was similar, although the radicle growth of seeds under osmotic stress was lower than under matric stress, what could explain the lowest infestation of *Orobanche* sp. in regions characterized by saline soil. The data in this study will be useful to forecast infection of host roots by *O. crenata*.

## Introduction

The holoparasitic weed *Orobanche crenata* Forsk. is responsible for important crop losses across the Mediterranean and West Asia where it parasitizes mainly Fabaceae species such as faba bean (*Vicia faba*), grasspea (*Lathyrus sativus*), lentil (*Lens culinaris*), pea (*Pisum sativum*), and vetches (*Vicia* sp.), but also Umbelliferae such as carrot (*Daucus carota*) (Grenz and Sauerborn, 2007; Parker, 2009). Because each *Orobanche* plant produces 1000s of minute seeds that persist viable in the soil for many years increasing the parasite seedbank in the soil and because infection and pathogenic process takes place underground (Joel et al., 2007) the effective control of this weedy species is extremely difficult (Rubiales et al., 2009).

*Orobanche* seeds germinate under favorable environmental conditions and the presence of chemical stimulants in the root exudates of proper plant species (Fernández-Aparicio et al., 2009). Before this, the *Orobanche* seeds are in an inactive state. Only after a conditioning period of several days that follows seed imbibition, *O. crenata* seeds can respond to germination stimulants (Van Hezewijk et al., 1993). However, this is not the case for other *Orobanche* species such as *O. cumana* and *O. aegyptiaca* (syn. *Phelipanche aegyptiaca*) in which this conditioning helps, but is not essential for stimulant receptivity (Plakhine et al., 2009). During the conditioning period, temperature (T), water potential (Ψ_t_), oxygen availability and growth regulators are known to affect the seed viability of several species of *Orobanche* and their germination response (Van Hezewijk et al., 1993; Kebreab and Murdoch, 1999; Gibot-Leclerc et al., 2004; Song et al., 2005).

Environmental factors, especially T and Ψ_t,_ affect the germination of conditioned seeds during incubation after exposure to germination stimulants (Kebreab and Murdoch, 2000). Once the parasitic seed germination is induced, an infective radicle arises from the seed coat and grows, following a positive gradient of germination stimulants until the host root, to which it can adhere and penetrate (Fernández-Aparicio et al., 2010). Therefore, the seeds, which show a large radicle, reach far roots what increases the infection efficiency. Temperature and Ψ_t_ can also affect the seed radicle elongation (Dodd and Donovan, 1999). Nevertheless, the impact of both T and Ψ_t_ on radicle elongation of *Orobanche* seeds are little understood. This information is essential for the development of germination and infection submodels, critical components to forecast effects of *O. crenata* on legume hosts.

Water potential quantifies the tendency of water to move from one point to another. In the soil, Ψ_t_ is mainly the sum of: (i) the gravitational potential (Ψ_g_); (ii) osmotic potential (Ψ_o_) as a consequence of the presence of ionic changes due to salts and non-ionically due to water binding by components on plant parts or other solutes; and (iii) matric potential (Ψ_m_) caused by water adsorption and surface tension phenomena in soil (Papendick and Campbell, 1980). Whereas Ψ_m_ and Ψ_o_ can change substantially and therefore affect seed germination, gravitational potential is determined solely by elevation of a point to some arbitrary reference point being negligible in near points (i.e., seed and adjacent water). In non-saline soils, Ψ_m_ is the dominant component (Papendick and Campbell, 1980; Chowdhury et al., 2011).

Furthermore, seed germination is differently affected by comparable Ψ_m_ and Ψ_o_, although their free energy measurements are equal (Hillel, 1972; Schmidhalter and Oertli, 1991). Thus, the seeds of different plants (Meiri, 1984; Schmidhalter and Oertli, 1991) and several microorganisms (Ramirez et al., 2004; Chowdhury et al., 2011) have been demonstrated to be more sensitive to low Ψ_m_ than low Ψ_o_. However, very little attention has been given to study the impact of Ψ_m_ and Ψ_o_ on seed germination of *Orobanche* species.

In this study, our objectives were to determinate the influence of temperature (T), water stress (Ψ_t_), and type of water stress (Ψ_m_ and Ψ_o_) on seed germination and radicle length of *O. crenata* during both the conditioning and the incubation periods. These results will be of value for development of predictive infection models.

## Materials and Methods

### *Orobanche* Seeds

Seeds were collected from *O. crenata* plants infecting faba beans during 2010 in Córdoba (37.51°N, 4.80°W, elevation of 110 m), southern Spain. Dry seeds were stored in glass containers in the dark at room temperature until use. Before use, seeds were disinfected with formaldehyde as described by González-Verdejo et al. (2005). To ensure that all germination requirements other than T and Ψ_t_ were satisfied, we included exogenous application of 1.2 mL of water or solution per 5-cm Petri dish, with 10 ppm GR24, a synthetic germination stimulant (Johnson et al., 1976) that was applied after conditioning period in the three experiments. In addition, we included seeds which were not exposed to GR24, which were incubated at 20°C at water potential value of 0 MPa, for assuring that this stimulant, is needed to seed germination in *O. crenata*. For each treatment, three replicate Petri dishes were used and the experiments were carried out twice. In all experiments, control seeds were conditioned with sterile distilled water.

### Water Potential Treatments

Because polyethylene glycol (PEG) solutions are relatively non-toxic to seeds (Song et al., 2005), aqueous solutions of PEG 8000 (Sigma 25322-68-3), or Milli-Q water were used for producing a range of matric potentials (0, −0.3, −0.6, −0.9, −1.2, and −3 MPa). The amount of PEG required for each combination T – Ψ_t_ was calculated using the polynomial equation of Michel and Kaufmann (1973) and revised by Michel (1983).

Likewise, sterile milli-Q water was modified osmotically by the addition of non-ionic glycerol (Panreac 56-81-5) to 0, −0.3, −0.6, −0.9, −1.2, and −3 MPa (Dallyn and Fox, 1980). The quantity of glycerol used to adjust the water activity (a_w_) of each solution was calculated using the Norrish’s equation (Harris, 1980) modified by Baeza et al. (2010). Finally, for a sample at given T, the Ψ_t_ was uniquely related to the a_w_ through the Kelvin equation:

(1) $$\Psi_{t}=\frac{RT_{k}}{M_{w}}Ln\,\;a_{w}\,\;\;\;\;\;\;\;\;\;\;\;\;\;\;\;\;\;\;\;\;\;\;\;\;\;\;\;\;\;\;\;\;\;\;\;\;\;\;\left(1\right)$$

where *R* is the universal gas constant, *T_k_* is Kelvin temperature and *Mw* is the molecular mass of water (Papendick and Campbell, 1980).

The Ψ_t_ of all solutions was then confirmed by measurement in a dewpoint potentiameter (WP4 Aqua Lab Water Meter, Decagon Devices, Pullman, WA, USA) and subjected to a slight adjustment when necessary.

### Effect of Temperature and Water Potential During the Conditioning Period

#### Experiment 1

Around 150 seeds of *O. crenata* were sown per 10-mm disks of glass fiber filter paper (WHATMAN 3645, 175 g m^−2^). Three disks (pseudoreplicates) were placed in a sterile 5-cm Petri dish lined with two layers of 50-mm diameter glass filter paper wetted with 1.5 mL of sterile milli-Q water or different conditioning media as described by Song et al. (2005). These media were PEG or glycerol solutions at −0.3, −0.6, −0.9, −1.2, and −3 MPa. The Petri dishes were sealed with Parafilm and wrapped with aluminum foil to provide absolute darkness. The Petri dishes were then placed in growth chambers at different temperatures (5, 10, 15, 20, 25, 30, and 35°C). After 5 days, other 0.6 mL of sterile water or conditioning medium was added to each Petri dish to maintain the Ψ_t_ and the dishes were placed back in the chambers for two more days.

After conditioning, the seeds were blotted to remove excessive water or conditioning media. Each disk from every replicate Petri dish were then transferred to a separate new 5-cm Petri dish containing two layers of filter paper wetted with 1.2 mL of sterile milli-Q water with 10 ppm GR24. Petri dishes were incubated at 20°C in the dark as describe above. Germination was examined under a compound microscope (Nikon Eclipse 80i; Nikon Corp., Tokyo) at 7 days after GR24 addition counting around 200 seeds per Petri dish. In addition, we randomly selected 30–40 germinated seeds per treatment and the length of their emerging radicle was also measured at this time. In total, 84 treatments [seven temperatures × six Ψ_t_ × two types of water stress (Ψ_m_ and Ψ_o_)] were evaluated. In all experiments, for each treatment, three replicate Petri dishes were used and the experiment was carried out twice.

#### Experiment 2

This experiment was nearly similar to our previous experiment but seed germination was assessed periodically at 2, 7, 10, 20, and 40 days after GR24 addition allowing calculation of seed germination percentage. Conditioning temperature was fixed to 20°C, maintaining the PEG and glycerol solutions at 0, −0.3, −0.6, −0.9, −1.2, and −3 MPa. In this case, between 0.1 and 0.3 mL of sterile water or conditioning medium was periodically added to each Petri dish to maintain the Ψ_t._

Because the germinated seeds were removed to measure the size of the radicles at 7, 20, and 40 days, this evaluation corresponds to seeds germinated between 0–7, 8–20, and 21–40 conditioning days (see Evaluation). Therefore, the radicle of seeds that germinated between the 10th to 20th days had 10 days to develop, and those that germinated between the 20th and 40th day had 20 days to develop. In total, the seeds were subjected to 12 treatments [six Ψ_t_ × two types of water stress (Ψ_m_ and Ψ_o_)] that were evaluated after five conditioning periods.

### Effect of Temperature and Water Potential During the Incubation Period

#### Experiment 3

This experiment was similar to *Experiment 1* except that *O. crenata* seeds were conditioned at 20°C in the dark for 10 days on the paper disks with sterile distilled water (160 μL per disk). Then the disks were blotted and transferred to new Petri dishes containing filter paper wetted with 1.2 mL of PEG or glycerol solution (0, −0.3, −0.6, −0.9, −1.2, and −3 MPa). Petri dishes were then incubated at 5, 10, 15, 20, 25, 30, and 35°C in the dark. The seed germination was evaluated at 7 and 10 days and the length of the emerging radicle of 30–40 seeds at 7 days as described above. In this case, 84 treatments [seven T × six Ψ_t_ × two types of water stress (Ψ_m_ and Ψ_o_)] were evaluated and the experiment was conducted twice.

### Evaluation

In all cases, the germination of *O. crenata* seeds were directly quantified on the Petri dishes using a magnification of 40× with the aid of a compound microscope (Nikon Eclipse 80i; Nikon Corp., Tokyo). For that, we counted the total of seeds of several fields of view that was taken at random. Seeds were considered germinated when the length of the emerging radicle was equal to or longer than its width. Length of the emerging radicle of seed was measured at a magnification of 200× with the aid of a compound microscope using the NIS-Element software (Nikon Corp., Tokyo, Japan).

### Statistical Analysis

Analysis of variance (ANOVA) was performed on germination percentage or radicle length depending on the design of each experiment. Both germination percentage and radicle length were log or arcsin-transformed when necessary for normality or homogeneity of variances. All experiments were repeated at once, and data from repetitions of each experiment were combined after checking for homogeneity of the experimental error variances by the *F* test (two variances). Because there were several interactions among independent variables (*Experiments 1 and 3*), to clarify the effects of Ψ_t_ (water vs. negative potentials) or type of water stress (osmotic vs. matric) on the dependent variables, we compared among them using orthogonal contrasts. When the type of water stress did not affect to type of water stress, ANOVA or regression analysis was performed on the whole of data using this variable as repetitions.

Linear regression analysis was used to evaluate the relationship between duration of conditioning period (days) and the cumulative germination percentage (*Experiment 2*). The duration of the conditioning period (days) was log-transformed. Various linear and non-linear regression models were evaluated for describing the effect of T and Ψ_t_ on seed germination and radicle length during the conditioning and the incubation periods (*Experiments 1 and 3*). The models tested were the generalized Analysis β model (Hau and Kranz, 1990), the Schödter angular model (Hau and Kranz, 1990), the Yin’s model (Yin et al., 1995) and several second- or third-order polynomial equations based on results of ANOVA analysis. We included the Ψ_t_ on the models as (Ψ*_t_* - Ψ*_tmin_*)^c^ when necessary. The used models were developed with an empirical approach according to the collected data. These models have been used to describe the influence of temperature and water stress on germination of broomrape seeds for the first time.

The Analysis β model (Hau and Kranz, 1990) was selected because it provided a good fit for all experiments and because each parameter has biological meaning. The Analysis β model uses the following equation:

(2) $$Y=k\times t^{a}\times {\left(1-t\right)}^{b}\times {\left(\Psi_{t}-\Psi_{t\,\min}\right)}^{c}\,\;\;\;\;\;\;\;\;\;\;\;\;\;\;\;\;\;\;\;\;\;\;\;\;\;\;\;\;\;\;\;\;\;\;\;\;\;\left(2\right)$$

in which *Y* = standardized germination percentage or radicle length that varied from 0 to 1 (*Y* = *G/G_max_* or *Y* = *RS/RS_max_*); *t* = standardized temperature [(*t* = (*T* -*T_min_*)/(*T_max_* -*T_min_*)]; Ψ*_t_* = water potential (0 ≥ Ψ*_t_* ≥ Ψ*_tmin_*); and *k*, *a*, *b*, and *c* are unknown parameters. *T_min_*, *_Tmax_*, and Ψ*_tmin_* were the minimum temperature, maximum temperature, and minimum *Ψ_t_* for seed germination, respectively. Maximum *Y* is reached when standardized *t* =*a/(a* + *b)*. Thus, for a given Ψ*_t_*, if the parameter *a < b* or *a*>*b*, the optimum temperature is shifted to the left of right, respectively. In this study, *T_min_* and *T_max_* were selected according to the data of our study, and the Ψ*_tmin_* used on the models (−2 MPa) was selected according previous experiments. A linear regression was applied to test the relationship between data estimated by non-linear regression and observed data. In all cases, the best regression model was chosen from many combinations of terms based on the significance of the estimated parameters (*P* ≤ 0.05), Mallow’s *Cp* statistic, Akaike’s information criterion modified for small data sets, the coefficient of determination (*R*^2^), *R*^2^ adjusted for degrees of freedom (*R_a_*^2^), and the pattern of residuals over predicted and independent variables.

## Results

Both T and Ψ_t_ affected the germination of *O. crenata* seeds during conditioning and incubation periods. Overall, the seeds germinated in a range of T between 10 and 25°C during conditioning and incubation periods, being germination strongly reduced at 25°C. On the contrary, no germination occurred at 5 or 35°C or under −3 MPa at any temperature and it was very limited at 30°C. For this reasons, equations of models were developed in all cases considering 5 and 35°C as *T_min_* and *T_max_*, respectively (**Table 1**). Moreover, there were several double and triple interactions among the independent variables depending on the experiments. No *O. crenata* seeds untreated with GR24 germinated.

**Table 1:** Effect of temperature (T), water potential (Ψ_**t**_), and type of water stress [matric (Ψ_**m**_) or osmotic (Ψ_**o**_)] during conditioning (*Experiment 1*) and incubation (*Experiment 3*) periods on seed germination and radicle length (0–1) of *Orobanche crenata* seeds according to the fitted Analysis β models.

Studied period	Y		Water stress	Analysis β model parameters^x,y^				*R^2^*	T opt (°C)


				*K*	*a*	*b*	*c*		
Conditioning	Gemination		Ψ_m_ and Ψ_o_^z^	365.071	5.433	4.332	1.016	0.986	18.9
Conditioning	Radicle		Ψ_m_	2.393	1.153	0.974	0.595	0.931	18.6
Conditioning	Radicle		Ψ_o_	1.588	1.214	1.027	1.222	0.841	18.5
Incubation	Gemination		Ψ_m_ and Ψ_o_^z^	3.253	2.032	1.479	1.736	0.941	19.5
Incubation	Radicle		Ψ_m_	3.436	1.872	0.821	0.5610	0.983	22.4
Incubation	Radicle		Ψ_o_	2.241	1.793	0.663	0.808	0.973	24.0

*^x^Seed gemination or radicle length (*Y*), temperature (T), and water potential (Ψ_*t*_) data were adjusted to a non-linear Analysis β model*Y*=*k* ×*t*^**a**^ × (1-t)^**b**^ × (Ψ_*t*_ -Ψ_*tmin*_)^*c*^ (Hau and Kranz, 1990), the equations of models were developed in all cases considering 5 and 30°C and −2 MPa as *T_*min*_*, *T_*max*_* and Ψ*_*tmin*_*, respectively.*

*^y^*Orobanche crenata* seeds were conditioned, before GR24 (conditioning period) addition, at 5, 10, 15, 20, 25, 30, and 35°C from −3 to 0 MPa (water) in humid chambers. Likewise, seeds were incubated, after the GR24 (incubation period), in the same conditions.*

*^z^There was not significant effect of type of water stress (matric or osmotic) on seed germination according to orthogonal contrast at *P* < 0.05.*

### Effect of Temperature and Water Potential During the Conditioning Period

#### Experiment 1

**FIGURE 1:**
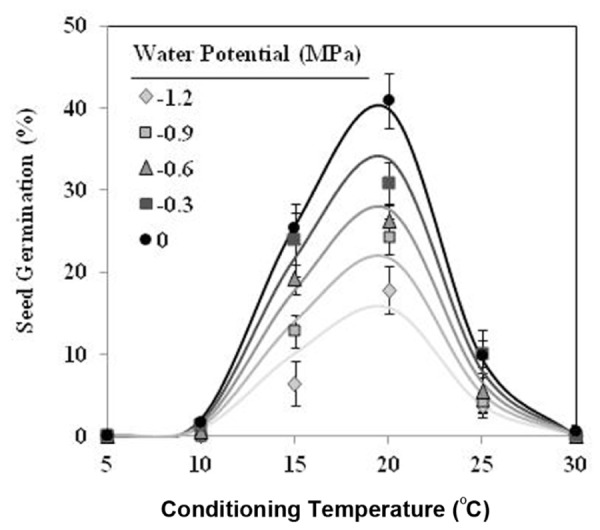
**Effects of temperature (°C) and water potential (MPa) on seed germination of *Orobanche crenata* during conditioning period**. The lines were fitted according to Analysis β equation (Hau and Kranz, 1990; **Table 1**). Points represent the average of 12 repetitions. Bars represent the SD of the mean.

Seeds of *O. crenata* conditioned in water potentials ≥−1.2 MPa germinated over the glass fiber filter papers at temperatures between 10 and 25°C. However, when conditioned at Ψ_t_ = −1.2 MPa the seeds did not germinate at 10°C. Maximum seed germination approached 41% in water at 20°C. The germination percentage of seeds conditioned in water was significantly (orthogonal contrasts, *P* = 0.035) higher than that of the seeds conditioned in negative water potentials. Conversely, the both matric and osmotic stress have similar (orthogonal contrasts, *P* = 0.6318) impact on the germination percentage of the seeds. For example, the mean of seed germination for the seeds conditioned at 20°C among −0.3 and −1.2 MPa under osmotic or matric stress were 25.9 and 23.7%, respectively. For that, the data from each type of water stress were used as independent repetitions to fit the models (**Table 1**). The fitted Analysis β model of Ψ_t_-T affecting the germination percentage is illustrated in **Figure 1**. The fitted model was highly significant (*P* < 0.001; *R^2^* and *Ra^2^* were >0.90) and the standardized residuals were randomly distributed over predicted values. The optimum T for maximum seed germination, obtained with fitted model, was 18.9°C. Thus, germination higher than 40% is only obtained with a conditioning at 17–20°C at water potential value of 0 MPa (**Figure 1**).

**FIGURE 2:**
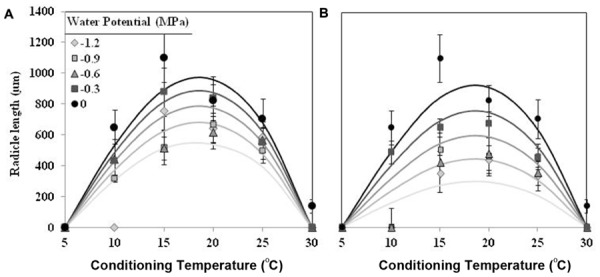
**Effects of temperature (°C) and matric **(A)** or osmotic **(B)** potentials (MPa) on radicle length of *O. crenata* seeds during conditioning period**. The lines were fitted according to Analysis β equation (Hau and Kranz, 1990; **Table 1**). Points represent the average of six repetitions. Bars represent the SD of the mean.

The effect of T on radicle length followed a similar trend as percentage of seed germination, although showing an optimum temperature lower. The largest radicle lengths were observed at 15 and 20°C for seed conditioned at water potential value of 0 MPa, which showed mean radicle lengths 1170 ± 75 μm and 826 ± 31 μm, respectively. For lower or higher temperatures the radicle lengths were shorter than previous one. Water potential (water vs. negative potentials) and the types (matric vs. osmotic stress) of water stress had significant (orthogonal contrast, *P* < 0.001) effect on radicle length of germinated seeds of *O. crenata*. Overall, the radicle length increased with increasing of Ψ_t_ from −1.2 to 0 MPa. Even so, the radicle length of seeds that were conditioned under osmotic stress (467 ± 11 μm) was significantly shorter (orthogonal contrast, *P* < 0.001) than those conditioned under matric stress (652 ± 12 μm). For this reason, two regression models were independently fitted for the data of each type of water stress. The Analysis β model showed an excellent fit for the radicle length data of both type of water stress (**Table 1**). The fitted models were highly significant (*P* < 0.001; *R^2^* and *Ra^2^* were >0.84) and the standardized residuals were randomly distributed over predicted values. The predicted optimum temperatures for maximum radicle length were around 18.5°C under both types of water stress. According to both fitted models, only the seed that were conditioned in water showed a radicle length over 900 μm (**Figures 2A,B**; **Table 1**).

#### Experiment 2

**FIGURE 3:**
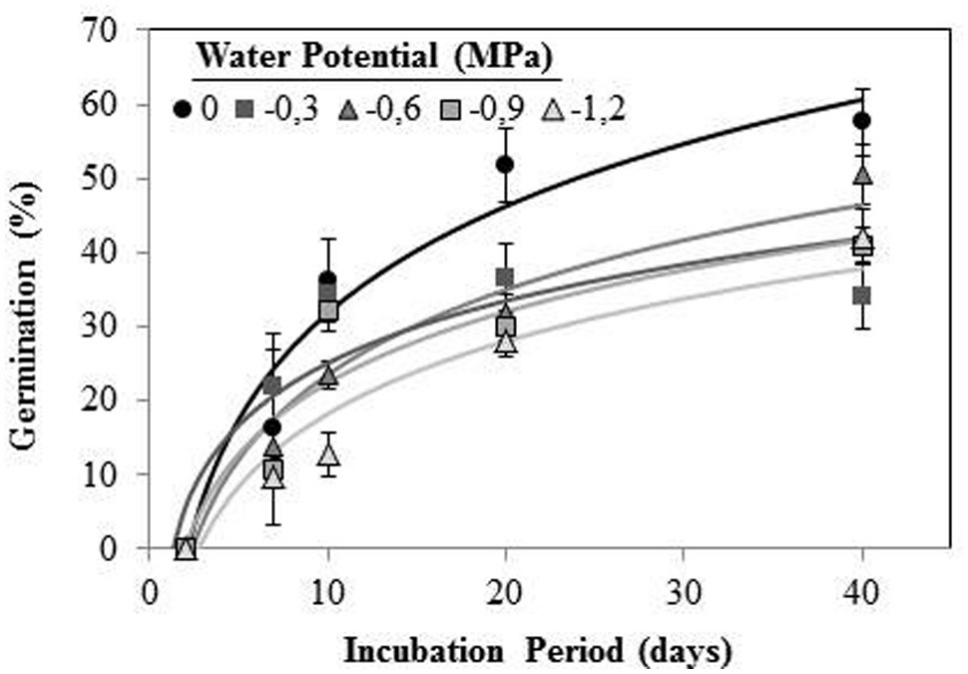
**Cumulative germination percentage of *O. crenata* conditioned at 20°C under different water potentials (MPa) during 40 days**. The lines were fitted according to logarithmic equation [G (%) =*a* +*log* (*days*)] for each water potential. Points represent the average of 12 repetitions. Bars represent the SD of the mean.

*Orobanche crenata* seeds conditioned at 20°C and Ψ_t_ ≥−1.2 MPa during 40 days, germinated under both types of water stress. No seeds geminated when they were conditioned at −3 MPa. Maximum germination (57.60 ± 4.5%) was obtained for seeds conditioned in water at the end of experiment (**Figure 3**). The germination percentage was significantly (orthogonal contrasts, *P* = 0.022) lower at negative potentials than water, but there was not significant differences between both types of waster stress (orthogonal contrasts, *P* = 0.411). For this reason, we used the data from each type of water stress as repetitions to fit regression lines (**Figure 3**). The cumulative percentage germination increased log-linearly (*R^2^* = 0.706; *P* < 0.001) with increasing of length of the conditioning period from 0 to 40 days (**Figure 3**). The short conditioning period that resulted in seed germination was the 7 days, with a germination percentage of ≈18% for the seeds conditioned in water. The comparison among the linear regression lines of each Ψ_t_ (between −1.2 and 0 MPa) showed equality of variances (*P* = 0.715), with significant differences between elevations (*P* < 0.001) but not (*P* = 0.475) among slopes (*b* = the apparent rate of germination increase). Thus, significant differences were found in the line slopes of water potentials 0 and −1.2 MPa (*P* < 0.05), which were significantly different (*P* < 0.05) to the remaining water potentials that formed a homogeneous group (*P*> 0.05; **Figure 3**).

**FIGURE 4:**
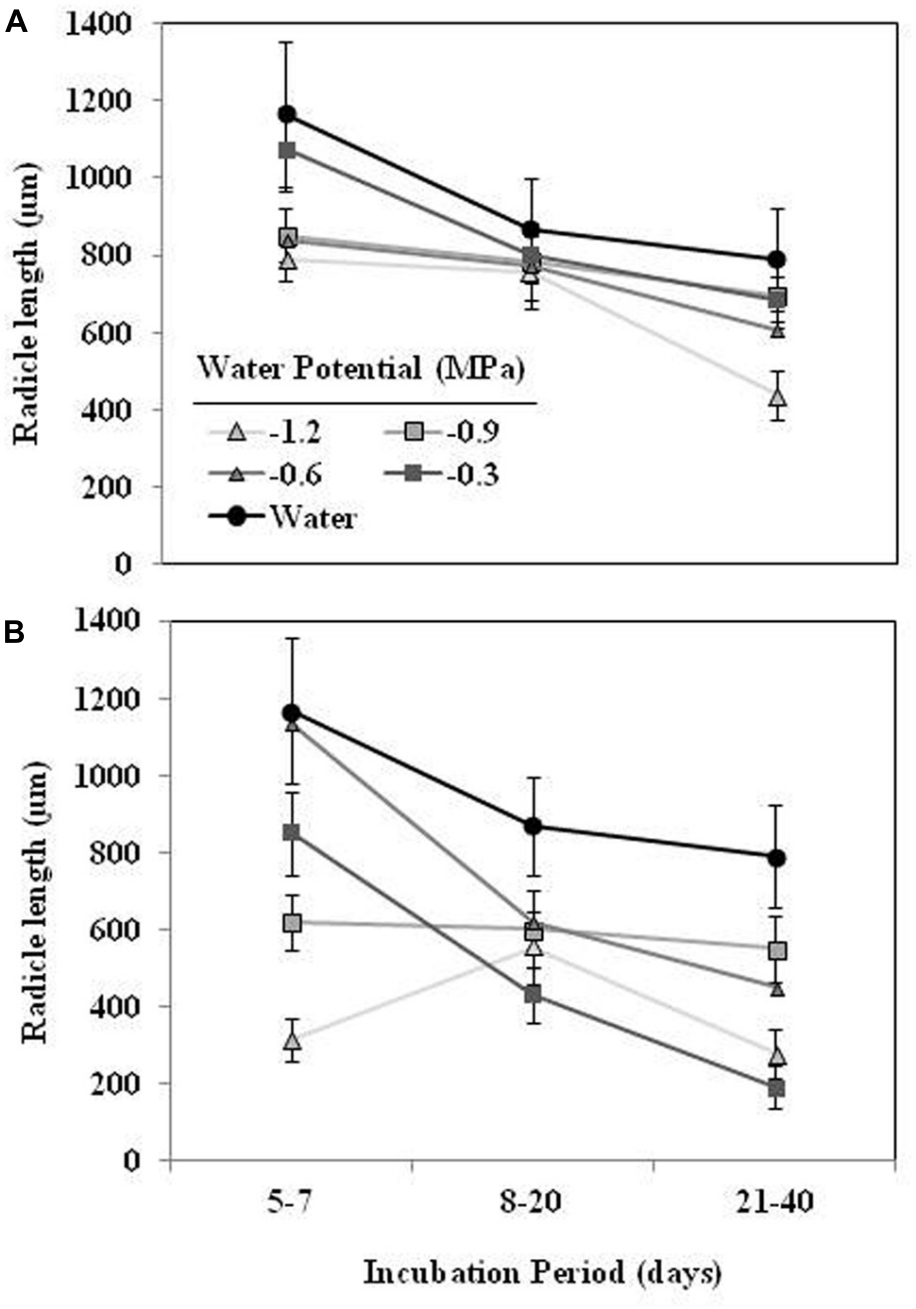
**Effect of the incubation period, during which the *O. crenata* seeds germinated (days after GR24 addition), on the radicle length seeds (μm) under matric **(A)** or osmotic potentials (B)**. Points represent the average of six repetitions. Bars represent the SE of each mean.

The mean radicle length of *O. crenata* seeds germinated in water during the first week ranged 1165 ± 68 μm (**Figure 4**). The effects of type of water stress, germination period and different interactions among independent variable (type of water stress-germination period and type of water stress-germination period-Ψ_t_) on the radicle length were significant (*P* < 0.05). The radicle length of the seeds conditioned in water (863 ± 28 μm) was higher (orthogonal contrast, *P* < 0.001) than radicle length of seeds (605.2 ± 9.5 μm) conditioned in negative water potentials. Moreover, the type of water stress also had significant (*P* < 0.001) effect on the radicle length being 725 ± 13 and 484 ± 11 μm under matric and osmotic stress, respectively. Overall, seeds germinated during the first days of the conditioning period showed a larger radicle than the later germinated ones. In the case of seeds conditioned in osmotic solutions, however, the seeds conditioned at −0.9 and −1.2 MPa showed a radicle length roughly constant (**Figures 4A,B**).

### Effect of Temperature and Water Potential During Incubation Period

#### Experiment 3

**FIGURE 5:**
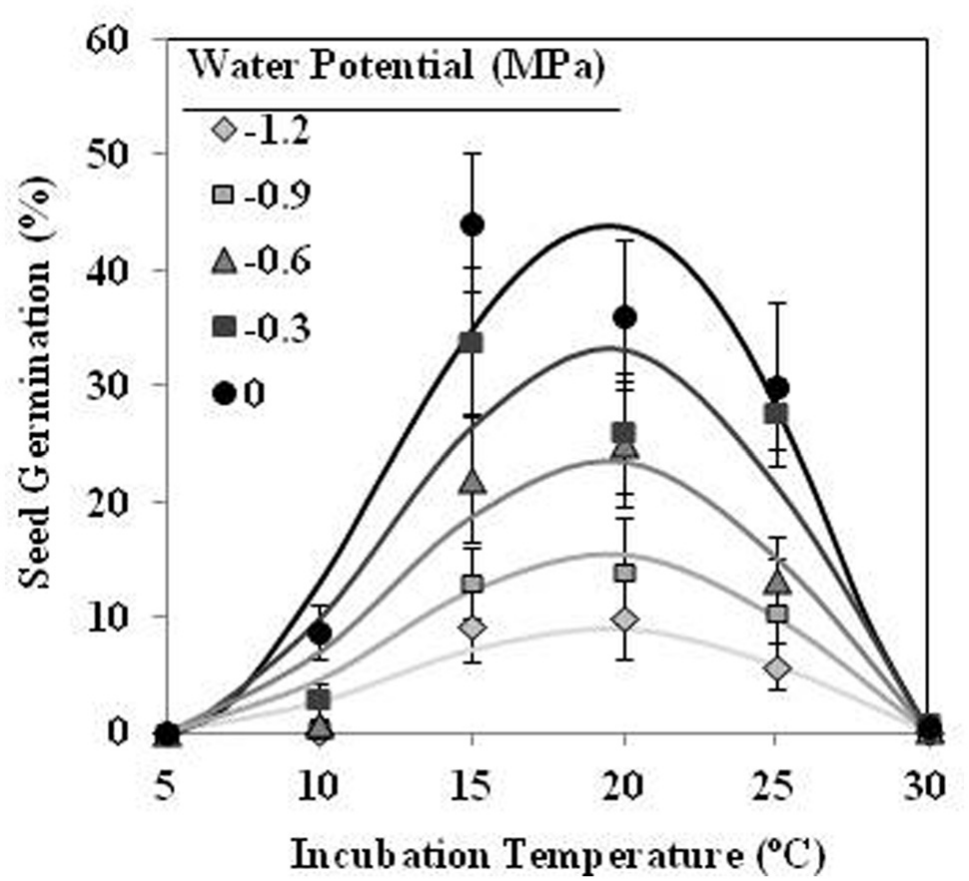
**Effects of temperature (°C) and water potential (MPa) on seed germination of *O. crenata* during incubation period**. The lines were fitted according to Analysis β equation (Hau and Kranz, 1990; **Table 1**). Points represent the average of 12 repetitions. Bars represent the SD of the mean.

The germination percentage of conditioned seeds increased with increasing incubation period from 5 to 10 days. The maximum germination percentage increased with the T between 10 and 20°C and then decreased between 20 and 30°C. Likewise, the final germination percentage increased with increasing Ψ_t_ from −1.2 to 0 MPa, approaching around 43% in water at 15°C (**Figure 5**). The germination percentage of conditioned seeds incubated in water was significantly higher (orthogonal contrasts, *P* < 0.001) than other incubated at negative potentials. On the contrary, there was no significant (orthogonal contrasts, *P* = 0.107) differences on the maximum germination percentage of conditioned seeds that incubated under matric or osmotic stress. The Analysis β equations (**Table 1**; **Figure 5**) fitted satisfactorily to the data of final germination percentage at each T-Ψ_t_ combination (*P* < 0.001; *R^2^* and *Ra^2^*> 0.93). The standardized residuals were randomly distributed over predicted again. The obtained-optimum T for seed germination was 19.5°C (**Table 1**; **Figure 5**).

**FIGURE 6:**
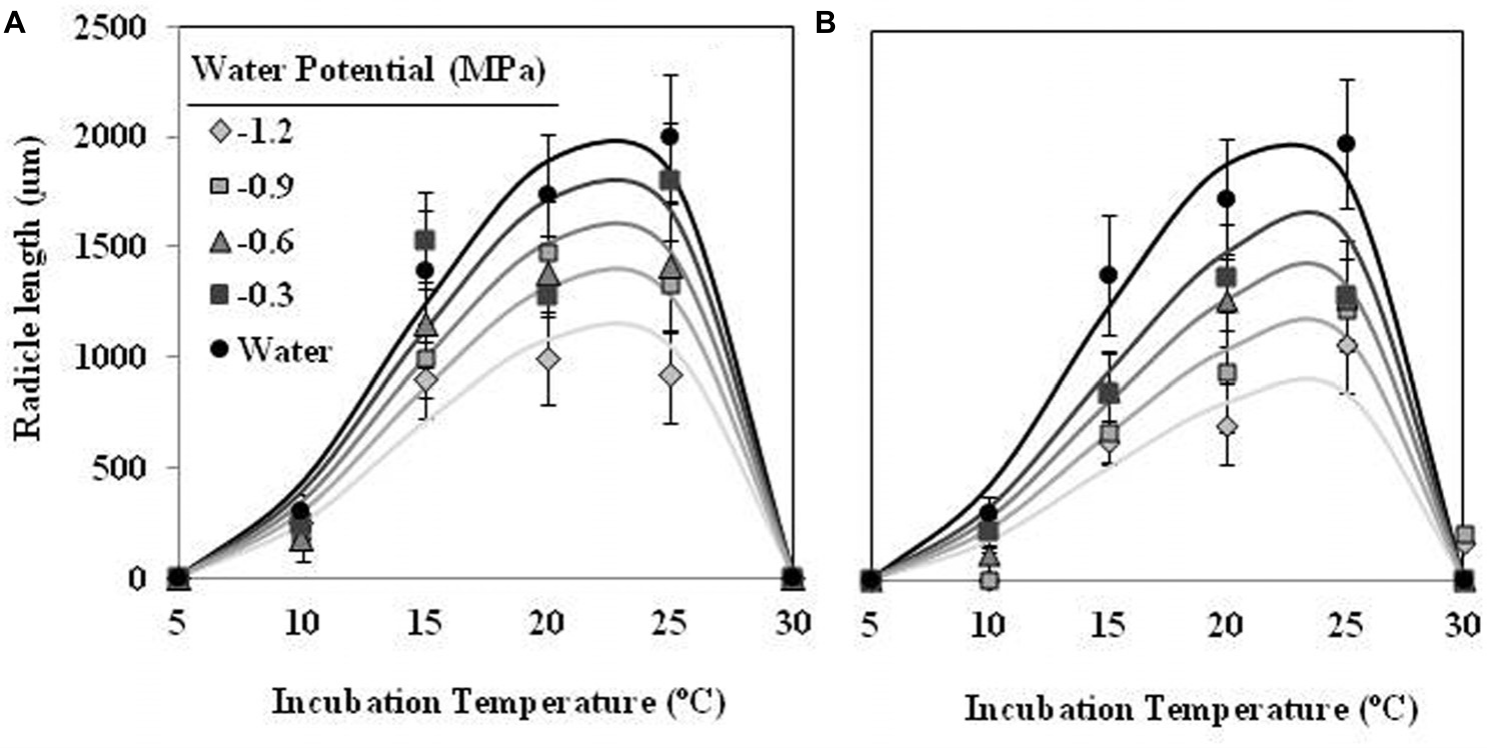
**Effects of temperature (°C) and matric **(A)** or osmotic **(B)** potentials (MPa) on radicle length of *O. crenata* seeds during incubation period**. The lines were fitted according to Analysis β equation (Hau and Kranz, 1990; **Table 1**). Points represent the average of six repetitions. Bars represent the SD of the mean.

Radicle length of seeds of *O. crenata* was highly affected by Ψ_t_ and T during the incubation period. For example, in the case of seeds incubated in water, average radicle length increased gradually from 304 μm at 10°C until a maximum of 1991 μm at 25°C (**Figure 6**). In addition, the conditioned seeds, which were incubated in water, showed higher (orthogonal contrasts, *P* < 0.001) radicle length than other incubated at negative water potentials. The type of water stress had too significant (orthogonal contrasts, *P* < 0.001) effect on the radicle length of seeds, being higher (1150 ± 588 μm) in the seeds incubated in PEG solutions than other (975 ± 498 μm) incubated in glycerol solutions. The curves describing the effect of Ψ_t_ and T on the radicle length of *O. crenata* seeds fitted satisfactorily (*P* < 0.001; *R^2^* and *Ra^2^* > 0.95) for each type of water stress (**Figures 6A,B**). The obtained optimum temperatures for radicle growth were 22.3 and 24.5°C under matric or osmotic stress, respectively (**Figures 6A,B**; **Table 1**). The same results were obtained when we studied the germination percentage at seven incubation days.

## Discussion

It has widely acknowledged that germination of *Orobanche* sp. seeds is influenced by environmental and microbiological factors, including T, Ψ_t_ (Kebreab and Murdoch, 1999, 2000; Song et al., 2005) as well as by microbe interactions in the rhizosphere (Mabrouk et al., 2007; Fernández-Aparicio et al., 2010). Traditionally, physiology-based models have been used to describe the effect of the environmental parameters on the germination of *Orobanche* seeds, although they have not been used to radicle length and have additional limitations. For example, the hydrothermal time model (Gummerson, 1986) requires daily evaluations for a good calculation of its rate of germination and it assumes that there is no interaction between Ψ_t_ and T. Kebreab and Murdoch (1999) proposed an alternative model to explain the interaction of Ψ_t_ and T, although it predicts that the seed population will eventually achieve 100% germination, which is not the case. To overcome this limitation, they later refined the model (Kebreab and Murdoch, 2000). Even so, hydrothermal time is currently the most used model to study seed germination of different weeds (Finch-Savage and Leubner-Metzger, 2006; Guillemin et al., 2013). Here we studied the effects of T, type of water stress (matric or osmotic) and Ψt on seed germination and radicle length of *O. crenata* seeds before and after exposure to GR24 as necessary exogenous stimulus for *O. crenata* germination in the absence of an appropriate host. For this purpose, we used the Analysis β model (Hau and Kranz, 1990) that has a series of advantages: (i) it provided an excellent fit for germination of *O. crenata* seeds after conditioning period and during the incubation period; (ii) it showed a good fit for radicle length of the seeds; and (iii) its parameters *T_min_*, *T_max_*, and Ψ*_tmin_* have biological significance, although it is mainly empirical model. Even so, empirical approach may be satisfactory for ecological modeling of seed germination (Forcella et al., 2000). Furthermore, mechanistic risk models can be easily developed considering the normalized rates of seed germination and radicle growth during conditioning and incubation periods using the Analysis β model. To develop mechanistic models, the main steps of the parasite life cycle *Orobanche* sp. (i.e., germination, radicle elongation to the host root, penetration, establishment, and plant develop) can be organized in a relational diagram according to the principles of the “systems analysis” and considering the normalized rates of the these steps (Leffelaar and Ferrari, 1989).

As a new feature, we studied separately the impact of both matric and osmotic stresses on seed germination of this parasitic plant due to little attention that has been paid to the differences between both stresses. For example, different authors have considered that the water stress caused by PEG solutions is of osmotic type (Kebreab and Murdoch, 2000; Song et al., 2005); although it has been previously shown that the water potential generated by PEG is predominantly (99%) due to matric forces (Steuter et al., 1981).

During the incubation period, the maximum germination of *O. crenata* seeds was obtained at 18–21°C. These values are mainly within the optimal range of 15–20°C for germination of *O. crenata* seeds (Van Hezewijk et al., 1993; Kebreab and Murdoch, 1999, 2000; Song et al., 2005). Small differences in optimum temperature for seed germination could be due to genetic variation within and among populations of *O. crenata* that attack legumes in different geographic regions (Van Hezewijk et al., 1993). This is in agreement with the substantial diversity among *O. crenata* populations revealed by molecular analyses (Román et al., 2002). At the optimal temperature for conditioning (20°C), the percentage of germinated seed decreased at both type of water stress from 0 to −1.2 MPa, the latter which is near to permanent wilting point of soil that is reached at −1.5 MPa (Cassel and Nielsen, 1986). Conversely, the seed germination was totally prevented at Ψ_t_ of −2 MPa in previous experiments. Our results are in agreement with Kebreab and Murdoch (2000) who showed a reduction in *O. aegyptiaca* seed germination when the water potential decreases from 0 to −1.33 MPa. Conversely, Song et al. (2005) did not observe significant decrease from 0 to −1 MPa in *O. aegyptiaca* and *O. ramosa* seed germination; although both species showed a marked reduction in seed germination at −2 MPa. It is interesting to remark that for some species as *O. ramosa*, the duration of the conditioning period influences the Ψ_t_ effect. E.g., the percentage of seed germination of this species is close to zero when the seeds are conditioned at −2 MPa during 4 days, and it is around 77% for the seed conditioned during 20 days (Gibot-Leclerc et al., 2004).

Even though the radicle elongation of seeds is an essential step in the parasite life cycle of *Orobanche* species, it has been scarcely studied when compared with seed germination. These few studies have focused on the effect of environmental and microbiological factors on radicle elongation. E.g., Westwood and Foy (1999) observed that radicle of *Orobanche* seeds are more sensible to nitrogen in ammonium form than nitrate. Barghouthi and Salman (2010) identified potential Biological Control Agents (BCAs), mainly *Pseudomonas* and *Bacillus* species, which adversely affected radicle elongation of *O. aegyptiaca* and *O. cernua*. The inhibition of radicle elongation of *Orobanche* seeds have also been identified as a resistance mechanism of red clover (*Trifolium pratense*) that is activated on plants treated with salicylate (Kusumoto et al., 2007). *Orobanche* radicle growth inhibition has also been reported by a number of plants and fungal metabolites (Fernández-Aparicio et al., 2013; Cimmino et al., 2014, 2015). In our study we found differences in the radicle length of *O. crenata* seeds in response to T, Ψ_t_ and type of water stress during conditioning period. Radicle length was maximum in the treatment of 15°C/0 MPa. In addition, at a given Ψt, radicle length was more sensitive to changes in osmotic than in matric potential. The effect of low osmotic potentials on seed germination and radicle elongation of *O. minor* during conditioning and incubation periods has been observed when using NaCl solutions; although the reduced radicle elongation could also be due to the toxic effect of ions on seeds (Hassan et al., 2010). In previous reports, matric stress exerts a more negative effect than osmotic stress on germination and seedling growth of different plants such as carrot (Schmidhalter and Oertli, 1991), bean (Meiri, 1984), pepper, and cotton (Shalhevet and Hsiao, 1986). These results may be explained by the fact that *O. crenata* seeds make up for the water stress in different ways and depending on the type of stress. For example, the plants can easily adjust their Ψ_t_ using the solutes under a saline medium, while they are less effective reducing the Ψ_t_ under matric stress due to a high metabolic energy requirement (Schmidhalter and Oertli, 1991). Likewise, fungi are able to reduce their Ψ_t_ by increasing the concentration of total sugar alcohols, although the patterns of accumulation of sugar alcohols change depending on the type of water stress (Ramirez et al., 2004). In addition, at low Ψ_o_, fungi are able to take up solutes to reduce their internal osmotic potential, an unavailable option when the Ψ_t_ is mainly matric (Jones et al., 2011).

In our experiments, percentage of seed germination increased logarithmically with the length of conditioning period during 40 days. This is concordant with the observation that *O. crenata* seeds reach maximum germination after a period of conditioning of 18–21 days (Van Hezewijk et al., 1993; Kebreab and Murdoch, 2000). Nevertheless, we did not distinguish clearly the secondary dormancy (wet dormancy) of the *O. crenata* seeds, i.e., a decreased in germination percentage after 21 or 49 days of conditioning at 20 and 10–15°C, respectively, as it has been observed for this species by Van Hezewijk et al. (1993). According to Kebreab and Murdoch (2000), *O. crenata* seeds, however, showed similar germination percentages when they were conditioned at 20°C during 20–40 days, and they needed more than 70 conditioning days to enter in a state of secondary dormancy. The induction of secondary dormancy at low temperatures during winter, might explain the decline in *Orobanche* infection observed by farmers in the case of late sowing (Parker and Riches, 1993). In addition, we have observed that seeds, that need more conditioning time to germinate, show the smallest radicles. The latter might also lead to a decline in infection rate of the crop.

During incubation period, *O. crenata* seeds germinated in a similar range of temperatures (10–25°C) than that which was required during conditioning, and the thermal optimum was the same (about 20°C). Similar optimum temperatures have been described for this species (Van Hezewijk et al., 1993), *O. aegyptiaca* (Jain and Foy, 1992; Kebreab and Murdoch, 2000), and *O. ramosa* (Gibot-Leclerc et al., 2004). Likewise, the germination percentage decreased with decreasing Ψ_t_ from 0 to −1.2 MPa, with no apparent differences between the types of water stress. According to our data, at given temperature (20°C), the percentage of germination of *O. crenata* seeds during conditioning period decreased at 16.4% per MPa, whereas the germination declined a 22.4% per MPa during the incubation. This fact suggests that *O. crenata* seeds appear more sensitive to low levels of Ψ_t_ during conditioning period than during the subsequent incubation phase. Water stress may be more limiting in the conditioning period, during which it is necessary that water enters into the seeds, than during the incubation period, when the seeds are already hydrated (Finch-Savage and Leubner-Metzger, 2006). The radicle length of *O. crenata* seeds was shorter of the seed incubated in osmotic than matric potentials, which has been previously discussed. This fact, the high sensibility of radicle elongation to osmotic stress could be related with the lowest infestation of *Orobanche* sp. in regions characterized by saline soil, as the region south to the Dead Sea in Jordan (Abu Irmaileh, 1998).

In summary, the results of this study clearly show that low matric and low osmotic potential had negative impacts on seed germination and radicle length of *O. crenata* seeds. At given Ψ_t_, the reduced percentage of seed germination was similar under matric and osmotic stress during the conditioning or incubation period. In contrast, our results show that low Ψ_o_ had a stronger negative effect on radicle length of *O. crenata* seeds than low Ψ_m_ during both periods.

## Conflict of Interest Statement

The authors declare that the research was conducted in the absence of any commercial or financial relationships that could be construed as a potential conflict of interest.
